# Isolated systolic hypertension and insulin resistance assessment tools in young and middle-aged Chinese men with normal fasting glucose: a cross-sectional study

**DOI:** 10.1038/s41598-021-04763-x

**Published:** 2022-01-14

**Authors:** Qing Gu, Jian Meng, Xue Hu, Jun Ge, Sui Jun Wang, Xing Zhen Liu

**Affiliations:** 1 Department of Endocrinology, Shidong Hospital, Shidong Hospital Affiliated to University of Shanghai for Science and Technology, No. 999, Shiguang Road, Yangpu District, Shanghai, 200438 China; 2 Hangzhou Aeronautical Sanatorium for Special Service of China Air Force, No. 27, Yang Gong Di, Xihu District, Hangzhou, 310007 Zhejiang China

**Keywords:** Cardiology, Endocrinology

## Abstract

The vital role of insulin resistance (IR) in the pathogenesis of isolated systolic hypertension (ISH) has been expounded at the theoretical level. However, research on the correlation between some specific IR indicators and ISH is still rare, especially at different glycemic statuses. We conducted this study to explore the association between three IR indicators and ISH among young and middle-aged adults with normal fasting plasma glucose (NFG). This large cross-sectional study included 8246 young and middle-aged men with NFG and diastolic blood pressure < 90 mmHg. The homeostasis model assessment for IR (HOMA-IR) index, triglyceride glucose (TyG) index, and the metabolic score for IR (METS-IR) were calculated with the corresponding formula. The proportions of ISH among young and middle-aged men were 6.7% and 4.4%, respectively. After fully adjusting, only HOMA-IR rather than TyG and METS-IR was significantly associated with ISH. Moreover, fully adjusted smooth curve fitting showed that the association between HOMA-IR and ISH were approximately linear in both two age groups (*P* for non-linearity were 0.047 and 0.430 in young and middle-aged men, respectively). Among young and middle-aged men with NFG, using HOMA-IR instead of noninsulin-dependent IR indicators may have advantages in the hierarchical management of ISH. Further longitudinal research may be needed to determine their potential causal relationship.

## Introduction

Isolated systolic hypertension (ISH) is defined as systolic blood pressure (SBP) ≥ 140 mmHg and diastolic blood pressure (DBP) < 90 mmHg. ISH is the most common hypertension phenotype in elderly individuals, which is mainly attributed to the increasing arterial stiffness with age^1^. However, ISH is also not uncommon among young and middle-aged individuals^2,3^. Unlike ISH in the elderly, the pathophysiological mechanism of ISH in the non-elderly population is more complex and heterogeneous, and may include increasing plasma volume, sodium reabsorption, insulin resistance (IR), and sympathetic nervous activity^4–6^. Therefore, recognition of IR in young and middle-aged adults with ISH may be of substantial clinical importance for its stratified management^7,8^.

However, in clinical practice, IR status is not easily determined. The homeostasis model assessment for IR (HOMA-IR) index, which employs fasting plasma glucose (FPG) and insulin, is the most commonly used IR evaluation tool^9^. However, fasting insulin testing is quite cumbersome, and simpler tools are sought. Recently, some noninsulin-based equations, such as the triglyceride glucose (TyG) index^10^ and metabolic score for IR (METS-IR)^11^, have been proposed as simple surrogates of IR.

Although several studies have shown that HOMA-IR, TyG, and METS-IR are closely associated with hypertension^12,13^, to the best of our knowledge, no studies have focused on their correlation with ISH among young and middle-aged adults, especially in the context of varying FPG levels. Thus, this study was conducted to examine the associations of HOMA-IR, TyG, and METS-IR with ISH among young and middle-aged adults with normal FPG (NFG).

## Results

### Clinical characteristics

The clinical characteristics of the study population stratified by ISH and nonhypertension are shown in Table 1. The study population consisted of 2415 young men and 5,831 middle-aged men, and their mean ages were 29.8 and 42.8 years, respectively. The proportions of ISH among young and middle-aged men were 6.7% and 4.4%, respectively.

**Table 1:** The basic clinical characteristics of young and middle-aged men with normal fasting glucose by blood pressure status.

Characteristics	Young men			Middle-aged men		
	Non-hypertension	ISH	*p* value	Non-hypertension	ISH	*p* value
No., n (%)	2252	163 (6.7)	–	5573	258 (4.4)	–
Age (year)	29.8 ± 3.8	29.9 ± 3.7	0.739	42.9 ± 4.6	42.5 ± 4.8	0.253
Smoking (%)	21.6%	19.3%	0.553	34.4%	26.7%	0.029
Drinking (%)	15.9%	17.6%	0.609	25.8%	29.3%	0.283
NAFL (%)	17.5%	20.8%	0.021	20.1%	24.2%	0.036
BMI (kg/m^2^)	23.5 ± 3.3	25.7 ± 4.4	< 0.001	24.0 ± 2.9	25.5 ± 3.2	< 0.001
WC (cm)	80.2 ± 8.7	85.2 ± 11.5	< 0.001	82.5 ± 7.5	85.8 ± 8.5	< 0.001
WHR	0.84 ± 0.05	0.86 ± 0.07	< 0.001	0.87 ± 0.05	0.89 ± 0.05	< 0.001
WHtR	0.46 ± 0.05	0.49 ± 0.07	< 0.001	0.48 ± 0.04	0.50 ± 0.05	< 0.001
SBP (mmHg)	120.2 ± 11.1	144.7 ± 5.1	< 0.001	118.3 ± 11.1	145.9 ± 5.9	< 0.001
DBP (mmHg)	72.1 ± 8.1	81.3 ± 5.6	< 0.001	74.7 ± 8.0	83.8 ± 4.8	< 0.001
HR (beats/min)	79.1 ± 12.2	85.3 ± 12.9	< 0.001	76.2 ± 11.0	82.2 ± 12.3	< 0.001
FPG (mmol/L)	5.17 ± 0.29	5.21 ± 0.25	< 0.001	5.20 ± 0.28	5.27 ± 0.29	0.001
UA (μmol/L)	389.9 ± 75.5	398.9 ± 78.4	0.143	381.2 ± 72.7	393.7 ± 79.3	0.007
TC (mmol/L)	4.50 ± 0.80	4.68 ± 0.78	0.006	4.77 ± 0.85	4.79 ± 0.96	0.651
TG (mmol/L)	1.30 ± 0.86	1.53 ± 1.12	0.002	1.71 ± 1.42	1.85 ± 1.21	0.113
HDLc (mmol/L)	1.42 ± 0.28	1.39 ± 0.33	0.141	1.39 ± 0.29	1.34 ± 0.27	0.006
LDLc (mmol/L)	2.47 ± 0.68	2.56 ± 0.70	0.122	2.64 ± 0.71	2.67 ± 0.79	0.520
ALT (U/L)	31.4 ± 25.3	36.1 ± 26.5	0.025	31.0 ± 25.2	36.4 ± 25.2	0.001
AST (U/L)	22.3 ± 10.5	23.9 ± 11.7	0.062	22.5 ± 11.7	24.1 ± 9.4	0.029
ALP (U/L)	67.7 ± 18.1	67.0 ± 16.9	0.661	67.5 ± 16.5	69.0 ± 16.7	0.168
GGT (U/L)	30.2 ± 23.4	35.4 ± 33.2	0.009	38.7 ± 36.2	46.1 ± 39.0	0.001
Insulin (μU/mL)	9.83 ± 5.95	12.57 ± 8.00	< 0.001	9.36 ± 5.14	11.73 ± 6.35	< 0.001
HOMA-IR	2.27 ± 1.38	2.92 ± 1.91	< 0.001	2.18 ± 1.22	2.75 ± 1.48	< 0.001
TyG	8.44 ± 0.51	8.56 ± 0.65	0.007	8.69 ± 0.55	8.79 ± 0.56	0.003
METS-IR	33.7 ± 6.3	37.6 ± 8.6	< 0.001	35.3 ± 6.0	38.1 ± 6.7	< 0.001

*NAFL* nonalcoholic fatty liver, *BMI* body mass index, *WC* waist circumference, *WHR* waist to hip ratio, *WHtR* waist to height ratio, *SBP* systolic blood pressure, *DBP* diastolic blood pressure, *HR* heart rate, *FPG* fasting plasma glucose, *UA* plasma uric acid, *TC* total cholesterol, *TG* triglyceride, *HDLc* high-density lipoprotein cholesterol, *LDLc* low-density lipoprotein cholesterol, *ALT* alanine aminotransferase, *AST* aspartate aminotransferase, *ALP* alkaline phosphatase, *GGT* gamma glutamyltranspeptidase, *HOMA-IR* homeostasis model assessment for IR index, *TyG* triglyceride glucose index, *METS-IR* metabolic score for IR.

Regarding age, men with ISH had higher anthropometric variables and HR, FPG, ALT, GGT, insulin, and IR indicators than nonhypertensive men. Young men with ISH had higher levels of TC and TG, but middle-aged men with ISH had higher UA and AST and lower HDLc than those nonhypertensive ones.

### Correlation between IR indicators and SBP

**Figure 1:**
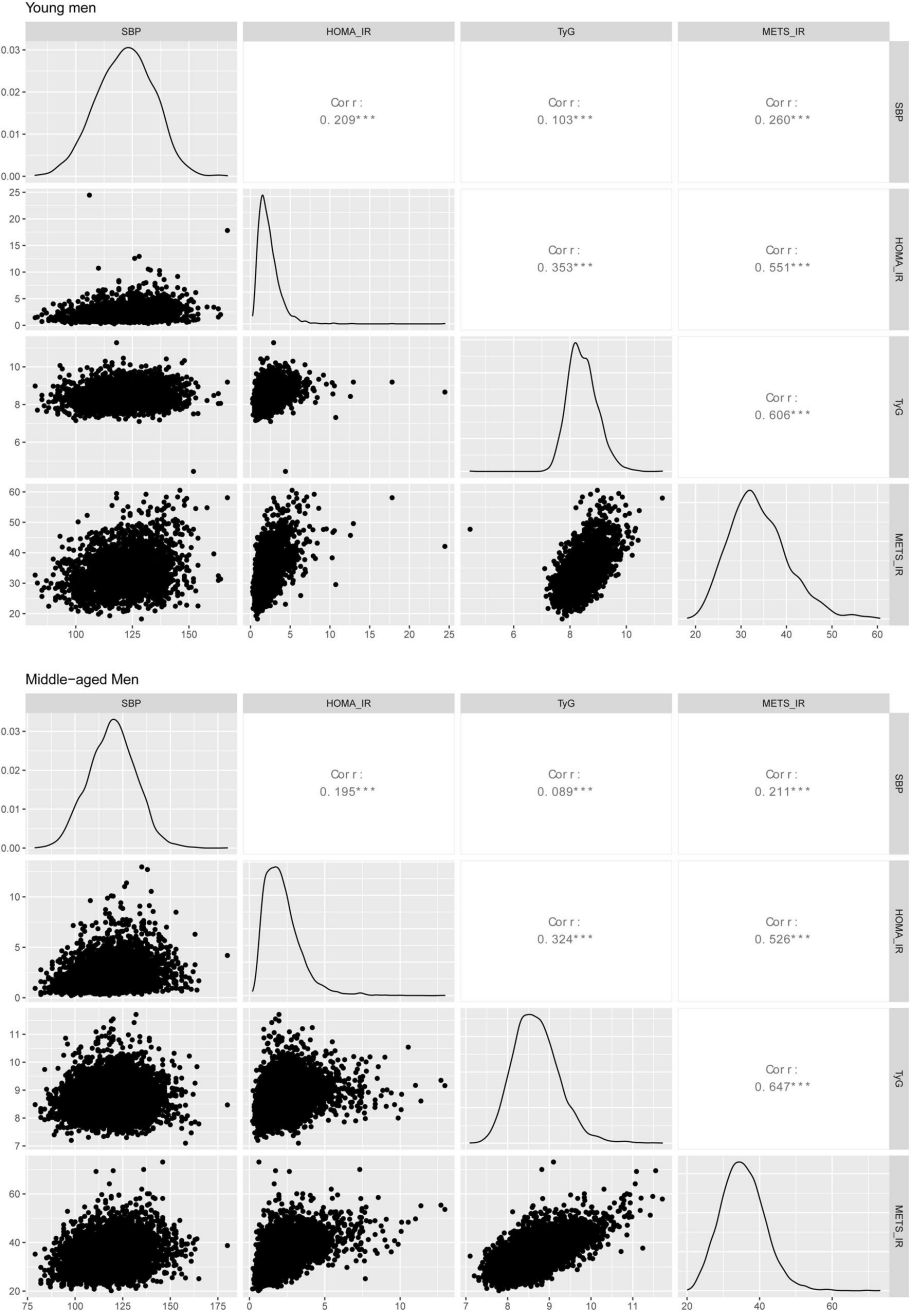
The correlation matrix between systolic blood pressure and insulin resistance indicators. *SBP* systolic blood pressure, *IR* insulin resistance, *HOMA-IR* homeostasis model assessment for IR index, *TyG* triglyceride glucose index, *METS-IR* metabolic score for IR.

As shown in Fig. 1, the correlation analysis showed that HOMA-IR, TyG, and METS-IR were significantly related to SBP in the two age groups; the correlation coefficients were 0.209, 0.103, and 0.260 in young men and 0.195, 0.089, and 0.211 in middle-aged men, respectively.

### Association of IR indicators with ISH

**Figure 2:**
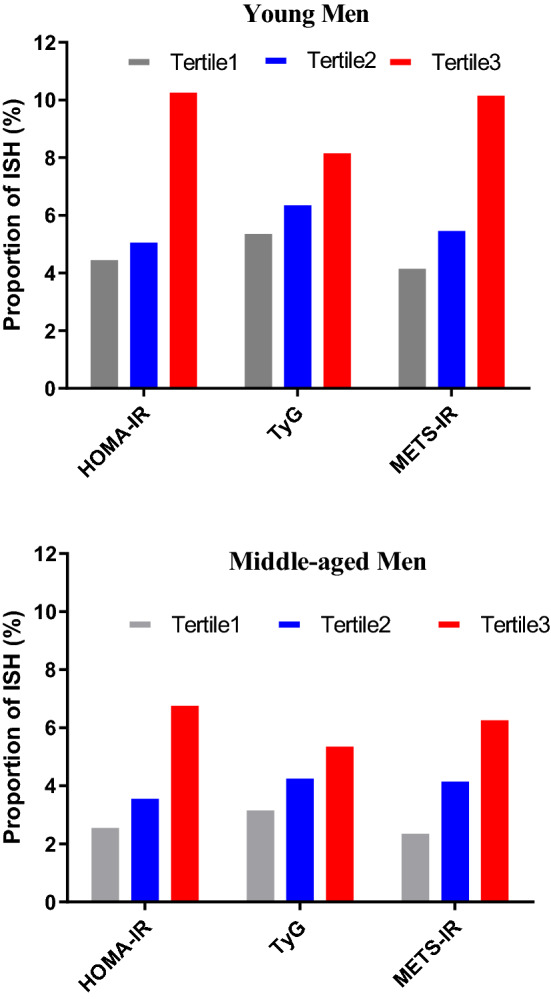
The proportion of ISH by categories of the tertiles of insulin resistance indicators. *ISH* isolated systolic hypertension, *HOMA-IR* homeostasis model assessment for IR index, *TyG* triglyceride glucose index, *METS-IR* metabolic score for IR, *T* tertiles.

We classified individuals into tertiles according to each IR indicator. The proportion of ISH from the bottom to the top tertile of IR indicators in both age groups were showing an upward trend. (Fig. 2).

The odds ratios (ORs) of the contribution of HOMA-IR, TyG, and METS-IR to ISH in the two age groups are shown in Table 2. In the unadjusted model (Model 1), all the three IR indicators were significantly associated with ISH. While in Model 2 (adjusted for abdominal obesity and NAFL) and Model 3 (Model 2 plus hyperuricemia, smoking and drinking), only HOMA-IR was significantly associated with ISH. The fully adjusted ORs (95% CI) of ISH were 2.369 (1.348–4.163) in young men and 1.757 (1.143–2.700) in middle-aged men for the top tertile compared with the lowest of HOMA-IR.

**Table 2:** Logistic regression analysis for the association of three IR indicators with ISH.

	Model 1			Model 2			Model 3		
	OR	LLCI	ULCI	OR	LLCI	ULCI	OR	LLCI	ULCI
**Young men**									
**HOMA-IR**									
T2 (1.57–2.54)	1.143	0.726	1.798	0.953	0.591	1.536	1.201	0.691	2.090
T3 (> 2.54)	2.418**	1.621	3.607	1.747*	1.082	2.819	2.369**	1.348	4.163
**TyG**									
T2 (8.17–8.64)	1.193	0.789	1.804	0.946	0.609	1.470	0.894	0.540	1.482
T3 (> 8.64)	1.572*	1.061	2.331	0.887	0.545	1.446	0.781	0.442	1.381
**METS-IR**									
T2 (30.68–36.18)	1.303	0.828	2.049	1.002	0.605	1.659	0.945	0.533	1.675
T3 (> 36.18)	2.529**	1.682	3.803	1.316	0.682	2.536	1.123	0.523	2.413
**Middle-aged men**									
**HOMA-IR**									
T2 (1.54–2.45)	1.355	0.944	1.945	1.104	0.756	1.612	1.081	0.701	1.667
T3 (> 2.45)	2.643**	1.910	3.656	1.788**	1.228	2.605	1.757*	1.143	2.700
**TyG**									
T2 (8.41–8.88)	1.343	0.967	1.865	0.950	0.669	1.348	0.753	0.502	1.131
T3 (> 8.88)	1.686**	1.231	2.311	0.913	0.626	1.333	0.800	0.517	1.237
**METS-IR**									
T2 (32.6–37.8)	1.747**	1.221	2.499	1.286	0.853	1.938	1.517	0.942	2.442
T3 (> 37.8)	2.659**	1.899	3.723	1.491	0.901	2.466	1.746	0.971	3.141

Model 1 was crude.

Model 2 was adjusted for abdominal obesity and non-alcoholic fatty liver.

Model 3 was adjusted for abdominal obesity, non-alcoholic fatty liver, hyperuricemia, smoking, and drinking.

*OR* Odds ratio, *LLCI and ULCI* lower level and upper level of 95% confidence interval, *T* tertile; the first tertile was used as the reference category, *HOMA-IR* homeostasis model assessment for IR index, *TyG* triglyceride glucose index, *METS-IR* metabolic score for IR; **< 0.01; *< 0.05.

**Figure 3:**
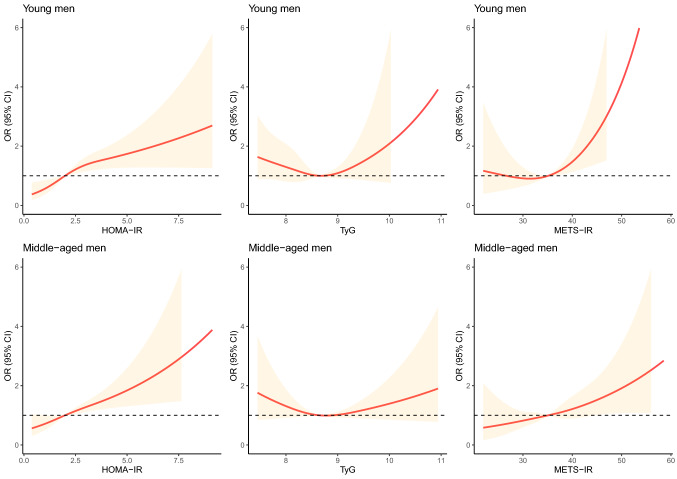
Fully adjusted association between insulin resistance indicators and ISH using cubic smoothing splines. *OR* Odds ratios, *ISH* isolated systolic hypertension, *HOMA-IR* homeostasis model assessment for IR index, *TyG* triglyceride glucose index, *METS-IR* metabolic score for IR; adjusted for abdominal obesity, non-alcoholic fatty liver, hyperuricemia, smoking, and drinking.

We then conducted a fully adjusted smooth curve fitting to visualize the potential non-linear associations of three IR indicators with ISH. As shown in Supplementary Fig. S1 (unadjusted) and Fig. 3 (fully adjusted), a approximate linear association of HOMA-IR with ISH were found (*P* for non-linearity was 0.047 in young men and was 0.430 in middle-aged men). The associations of TyG and METS-IR with ISH were easily affected by confounding factors.

## Discussion

This cross-sectional study mainly focuses on the associations of different IR indicators with ISH among young and middle-aged men with NFG. Our results showed that only HOMA-IR but not TyG and METS-IR was independently associated with ISH in young and middle-aged men with NFG. In addition, a approximative linear associations between HOMA-IR and ISH had also been found by smooth curve fitting.

The prevalence of ISH according to age is a typical J-shaped pattern, with a 2–8% frequency in 30-year-old individuals, and the nadir occurs at approximately age 50 (0.1–0.8%)^14,15^. In the present study, our results were also consistent with this pattern; that is, the proportion of ISH in young men was higher than that in middle-aged men (6.7% vs 4.4%). In individuals younger than 35 years, the prevalence of ISH is higher in men and negligible in women^14^.

The ISH trend in untreated adults is increasing. Data from the National Health and Nutrition Examination Survey indicate that the prevalence of untreated ISH in 18- to 39-year-old U.S. adults increased from 0.7% in 1988–1994 to 1.6% in 1999–2004 and then to 1.9% in 2005–2010^16,17^. Other studies have also validated the growth trend of ISH^18^. Lifestyle and nutrition changes and accompanying epidemics of obesity, especially visceral obesity^19^, in young and middle-aged adults are potential contributors to the increasing tendency of ISH in young and middle-aged adults^20^. In this study, we also observed that the men with ISH had higher anthropometric variables than nonhypertensive men in both age groups, which was consistent with the clinical characteristics of ISH in young and middle-aged adults reported in previous studies^16–18^.

Many studies have demonstrated the vital role of IR in the pathogenesis of hypertension in the general population with different BMIs and glycemic statuses^21–23^. Data from a 20-year follow-up study (including 3413 Americans ages 18–30 years) indicated that fasting insulin levels were positively associated with the incidence of hypertension, which is the strongest evidence thus far connecting IR associated with hypertension in young adults^24^. Regrettably, the long-term prospective cohort study did not further analyze the relationship between hypertension phenotypes and insulin levels.

To date, studies on the correlation between IR and ISH among young and middle-aged adults are still relatively rare. In the Olivetti Heart Study (n = 356, age < 50 years, and 3% ISH)^25^, Strazzullo et al. compared the HOMA-IR values between the ISH group and the normotensive group. However, due to the small sample size, the difference in HOMA-IR values did not reach statistical significance. When they lowered the diagnostic cutoff point of ISH (SBP ≥ 130 mmHg and DBP < 85 mmHg), which increased the proportion of ISH to 6%, a significant difference was observed. In the present study, we found that HOMA-IR was robustly associated with ISH in both young and middle-aged men with NFG. These results further highlight the role of IR in ISH among young and middle-aged adults, even in the context of normal blood glucose levels.

Actually, available evidence suggests that IR and compensatory hyperinsulinemia may play more prominent roles in the initiation of hypertension in the early stages of abnormal glucose metabolism than in the later stages^26^. In the early stages, IR can cause an increase in sodium reabsorption, renin secretion, and sympathetic nervous activity, which are mainly responsible for the increase in SBP^7,8,27^. With the chronic elevation of plasma glucose and insulin, advanced glycation end products and inflammation follow, which could promote atherosclerosis and cause resultant vascular resistance^28^. At this time, vascular resistance may be the main contributor to BP elevation^29^. Therefore, recognition of IR in young and middle-aged adults with NFG is of great significance to the prevention and management of ISH.

In clinical practice, the choice of IR evaluation tools is not an easy task and needs to consider factors such as sample size, budget, and glycemic status^30^. In fact, choosing more than one IR indicator in clinical practice if the budget allows is recommended and may help complementarity between IR indicators^31^. To provide more options for the monitoring and management of ISH among young and middle-aged adults. In addition to HOMA-IR, we also examined the association of two noninsulin-dependent IR indicators with ISH under normal blood glucose levels. However, we did not observe that TyG and METS-IR were significantly associated with ISH.

FPG is an integral part of TyG and METS-IR, so their ability to assess IR may be easily affected by blood glucose levels. In this study, we limited subjects to individuals with only normal blood glucose levels, which may partially explain the performance of TyG and METS-IR. Although FPG is also one of the components of HOMA-IR, the calculation of HOMA-IR also depends on the insulin level. Moreover, the measurement of insulin is also susceptible to glycemic status. For those with longer duration or uncontrolled diabetes, their increasingly depleted β-cell function has been unable to secrete enough insulin^32,33^. In contrast, the insulin level may be able to reflect the degree of IR more accurately in the early stages of impaired glucose metabolism^34^.

The major strengths of this study are that it can still guarantee a certain sample size after excluding women and individuals with abnormal glucose metabolism and DBP ≥ 90 mmHg. However, the present study is not without limitations. First, our data were extracted from the health screening database. So we lack the data on postprandial glucose levels or 75-g oral glucose tolerance, which prevents us from excluding those individuals with impaired glucose tolerance. Second, glycosylated hemoglobin or glycated albumin, indicators of long-term glycemic status^35,36^, are also not included in routine health screening items. Third, the causal association of these IR indicators with ISH in young and middle-aged men with NFG cannot be inferred due to the cross-sectional design.

In conclusion, our study indicated that HOMA-IR, rather than noninsulin-dependent IR indicators (TyG and METS-IR), was significantly associated with ISH among young and middle-aged men with NFG. This result indicates that HOMA-IR may be a potential tool for hierarchical management of ISH among them. Our findings not only further highlights the close relationship between IR and ISH in non-elderly adults, but also suggest that the influence of glycemic status needs to be considered when selecting IR indicators.

## Methods

### Study population

We collected data from Chinese adults who received general health screenings between January 2013 and July 2019 in the city of Shanghai and Hangzhou. They were all of Han nationality, from eastern China (mainly the Yangtze Delta region), and "apparently healthy" (without serious diseases). Because the proportion of ISH among non-elderly women was too small to perform regression analysis, we only took men as research subjects.

Inclusion criteria were ages 18–50, DBP less than 90 mmHg, and with NFG. Exclusion criteria were taking antihypertensive, hypoglycemic, and lipid-lowering medications. Finally, 8246 young and middle-aged men with DBP < 90 mmHg and NFG were eligible for this study. Approval for this study was obtained from the ethics committee of Shanghai Shidong Hospital (2021-KY016). Due to the retrospective and observational design of this study, we cannot obtain the informed consent of all subjects. The need for written informed consent from individual subjects was waived by the ethics committee of Shanghai Shidong Hospital. All research was performed in accordance with relevant guidelines and regulations.

### Data collection

Before starting the medical examination, basic information about the participants were collected, including family history, medical history, diseases, medications, smoking and alcohol consumption. The SBP, DBP, and heart rate (HR) were obtained 3 times on the right arm after at least 5 min of rest using an automatic BP monitor (HEM-1000, OMRON, Japan). Height and weight were measured with subjects barefoot and in light clothing on digital scales. Waist circumference (WC) and hip circumference (HC) were measured according to a standardized protocol and technique by well-trained nurses. Abdominal ultrasonography was undertaken by clinical radiologists using a 3.5 MHz probe.

Blood specimens were drawn from the antecubital vein in the morning after at least 8 h of overnight fasting. Measurements of FPG, plasma uric acid (UA), total cholesterol (TC), triglyceride (TG), low-density lipoprotein cholesterol (LDLc), high-density lipoprotein cholesterol (HDLc), alanine aminotransferase (ALT), aspartate aminotransferase (AST), alkaline phosphatase (ALP), and gamma glutamyl transferase (GGT) were taken using an automatic biochemical autoanalyzer (Advia 1650 Autoanalyzer; Leverkusen, Germany). Serum levels of insulin were measured by the electrochemiluminescence immunoassay method (Roche, Mannheim, Germany).

### Definitions

ISH was defined as having an SBP ≥ 140 mmHg and DBP < 90 mmHg, and nonhypertension was defined as SBP < 140 mmHg and DBP < 90 mmHg. NFG was defined as FPG less than 5.6 mmol/L. We stratified the subjects into young and middle-aged groups with the age of 35 as the dividing line. Body mass index (BMI) was calculated as weight divided by the square of height. WC divided by HP was the waist-to-hip ratio (WHR). WC divided by height provided the waist-to-height ratio (WHtR). Other formulas were as follows: HOMA-IR = [FBG (nmol/L) × insulin (μU/mL)/22.5]; TyG = Ln [fasting TG (mg/dL) × FPG (mg/dL)/2]^10^; METS-IR = Ln [(2 × FPG) + TG] × BMI)/(Ln[HDLc])^11^.

### Statistical analysis

All data were analyzed using SPSS 18.0 (SPSS Inc., Chicago, IL, USA) or R 4.0.5 (R Foundation for Statistical Computing, Vienna, Austria). Quantitative data are expressed as the means ± standard deviation (SD) and were compared using *t*-tests. Categorical variables were expressed as the number (%) and compared using the chi-square test. Correlation matrix diagram was used to show the distribution of IR indicators and the correlation with SBP (Pearson correlation).

We evaluated the association of HOMA-IR, TyG, and METS-IR with ISH using multivariate logistic regression analyses, which were adjusted for nonalcoholic fatty liver (NAFL), hyperuricemia, alcohol intake, smoking status, and abdominal obesity. Because the calculation of IR indicators uses lipid parameters and BMI, dyslipidemia and general obesity were not adjusted to prevent overadjustment. The three IR indicators were divided into three tertiles, and the lowest tertile (T1) was used as a reference category. Limits of the generated tertiles of the three IR indicators are shown in Table 2. Furthermore, a logistic regression with cubic spline functions and smooth curve fitting (restricted cubic spline) were used to examine the potential nonlinear association between the three IR indicators and ISH. A *P*-value < 0.05 was considered statistically significant.

### Ethics approval and consent to participate

The research protocol was approved by the ethics committee or review committee of the Shidong Hospital. Because the study was a retrospective study, there was no informed consent from the patients.

### Consent for publication

All authors agree to publish this work.

## Supplementary Information

Supplementary Figure S1.

Supplementary Legends.

## Data Availability

The datasets used and/or analyzed during the current study are available from the corresponding author on reasonable request.

## Abbreviations

IR: Insulin resistance
ISH: Isolated systolic hypertension
HOMA-IR: Homeostasis model assessment for IR index
METS-IR: Metabolic score for IR
TyG: Triglyceride-glucose index
