# Supplementary figures and images for: Isolated systolic hypertension and insulin resistance assessment tools in young and middle-aged Chinese men with normal fasting glucose: a cross-sectional study
Source: Sci Rep. 2022 Jan 14;12:758. doi: 10.1038/s41598-021-04763-x (PMC8760306; doi:10.1038/s41598-021-04763-x)

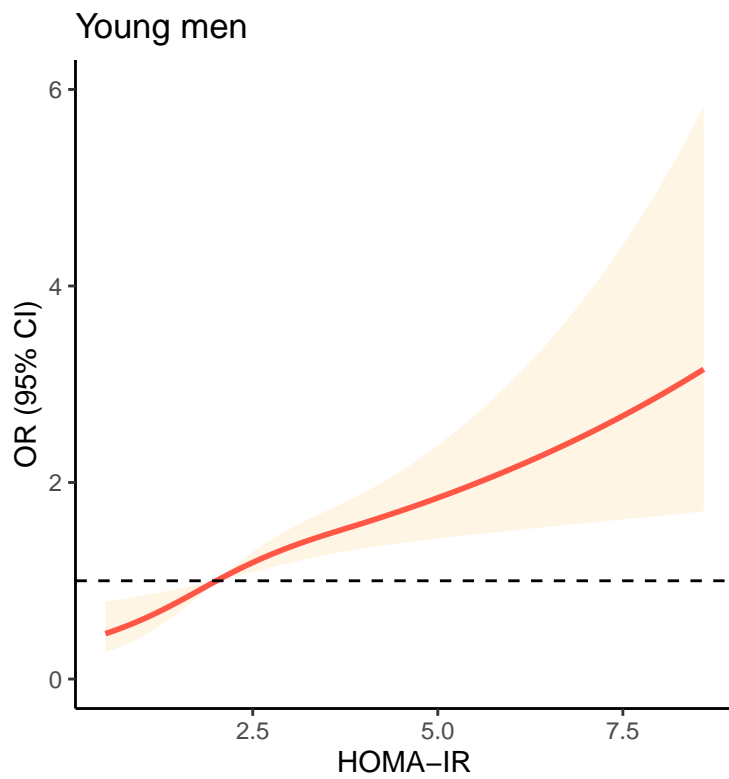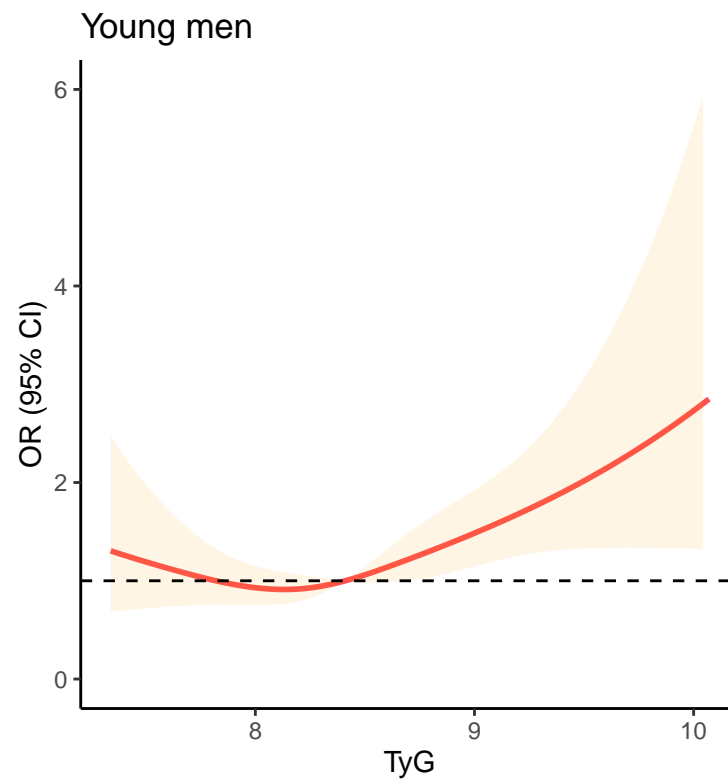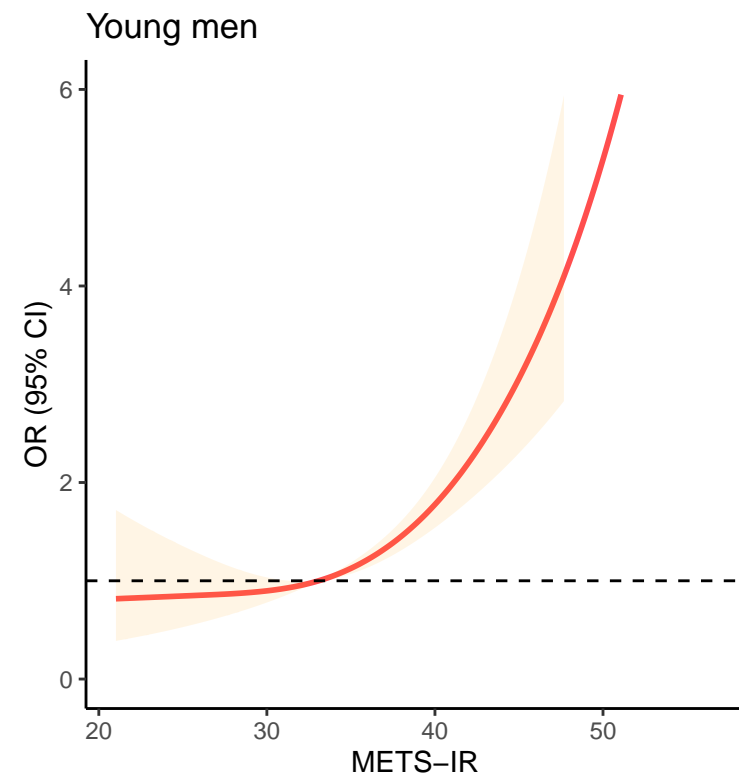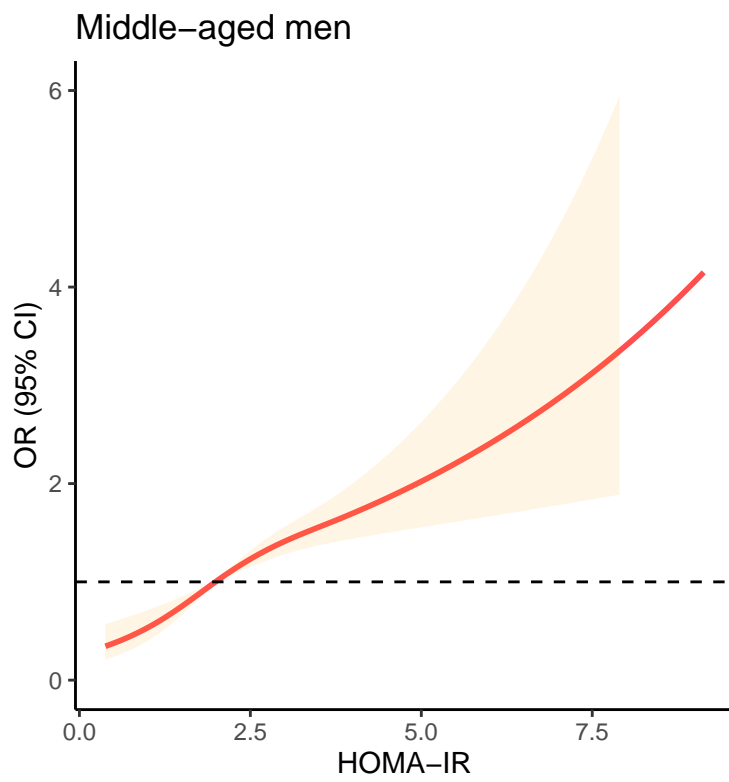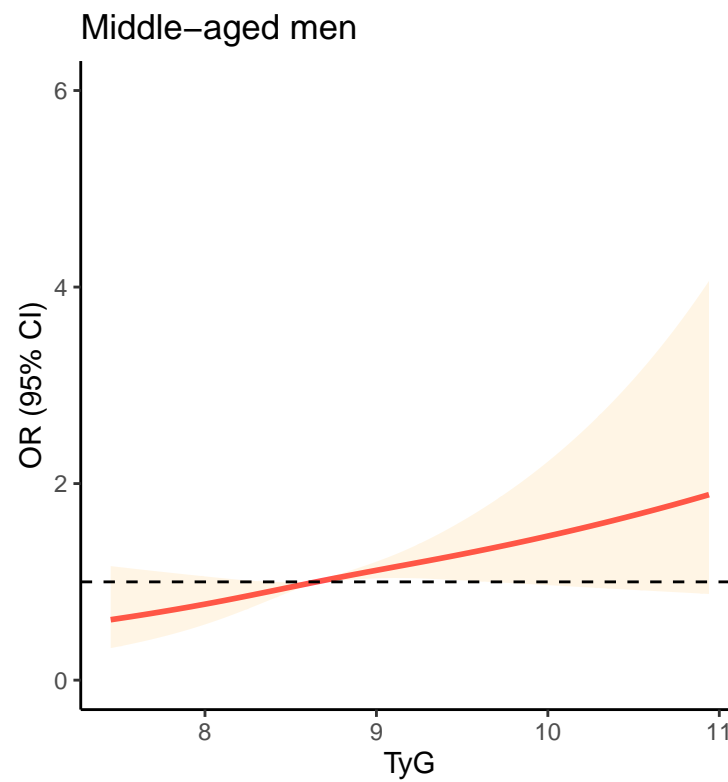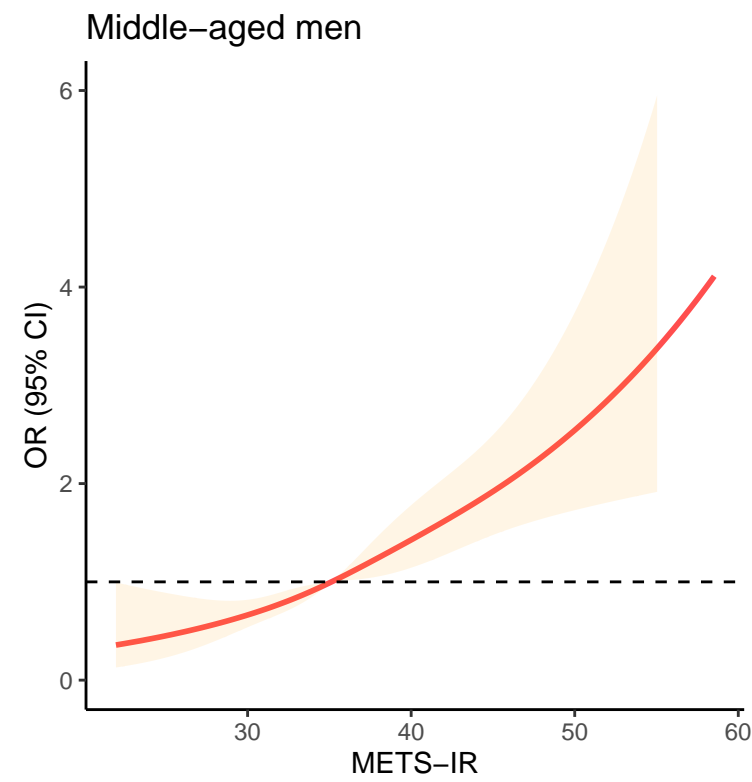

Supplement: Supplementary file 1 — Supplementary Figure S1. [file 41598_2021_4763_MOESM1_ESM.pdf]
