# Supplementary material for: Isolated systolic hypertension and insulin resistance assessment tools in young and middle-aged Chinese men with normal fasting glucose: a cross-sectional study
Source: Sci Rep. 2022 Jan 14;12:758. doi: 10.1038/s41598-021-04763-x (PMC8760306; doi:10.1038/s41598-021-04763-x)
Supplement: Supplementary file 2 — Supplementary Legends. [file 41598_2021_4763_MOESM2_ESM.docx]

**Figure S1.** **Unadjusted association between insulin resistance indicators and ISH using cubic smoothing splines.** Odds ratios, OR; ISH, isolated systolic hypertension; HOMA-IR, homeostasis model assessment for IR index; TyG, triglyceride glucose index; METS-IR, metabolic score for IR;
